# Bithionol blocks pathogenicity of bacterial toxins, ricin, and Zika virus

**DOI:** 10.1038/srep34475

**Published:** 2016-09-30

**Authors:** William Leonardi, Leeor Zilbermintz, Luisa W. Cheng, Josue Zozaya, Sharon H. Tran, Jeffrey H. Elliott, Kseniya Polukhina, Robert Manasherob, Amy Li, Xiaoli Chi, Dima Gharaibeh, Tara Kenny, Rouzbeh Zamani, Veronica Soloveva, Andrew D. Haddow, Farooq Nasar, Sina Bavari, Michael C. Bassik, Stanley N. Cohen, Anastasia Levitin, Mikhail Martchenko

**Affiliations:** 1 Keck Graduate Institute, Claremont, CA 91711, USA; 2 Foodborne Toxin Detection and Prevention Research Unit, Western Regional Research Center, United States Department of Agriculture (USDA), Albany, CA 94710, USA; 3 Department of Genetics, Stanford University School of Medicine, Stanford, CA 94305, USA; 4 US Army Medical Research Institute of Infectious Diseases (USAMRIID), Fort Detrick, MD, 21702, USA; 5 Henry M. Jackson Foundation, Bethesda, MD, 20817, USA; 6 Biotechnology High Performance Computing Software Applications Institute (BHSAI), Frederick, MD, 21702, USA

## Abstract

Diverse pathogenic agents often utilize overlapping host networks, and hub proteins within these networks represent attractive targets for broad-spectrum drugs. Using bacterial toxins, we describe a new approach for discovering broad-spectrum therapies capable of inhibiting host proteins that mediate multiple pathogenic pathways. This approach can be widely used, as it combines genetic-based target identification with cell survival-based and protein function-based multiplex drug screens, and concurrently discovers therapeutic compounds and their protein targets. Using B-lymphoblastoid cells derived from the HapMap Project cohort of persons of African, European, and Asian ancestry we identified host caspases as hub proteins that mediate the lethality of multiple pathogenic agents. We discovered that an approved drug, Bithionol, inhibits host caspases and also reduces the detrimental effects of anthrax lethal toxin, diphtheria toxin, cholera toxin, *Pseudomonas aeruginosa* exotoxin A, *Botulinum* neurotoxin, ricin, and Zika virus. Our study reveals the practicality of identifying host proteins that mediate multiple disease pathways and discovering broad-spectrum therapies that target these hub proteins.

In recent years, a better understanding of protein interaction networks has led to the identification of highly connected hub proteins and pathways that are commonly used by a number of different pathogens and in a range of diseases^1^. These hubs represent promising targets for drug development.

Most disease networks have the “small-world” property, where proteins are only a few interactions away from any other proteins^2^. Therefore, inhibiting a given node can potentially affect the state of most nodes in its vicinity as well as the activity of the network itself. In this way, therapeutic inhibition of nodes and hubs within one disease network can affect other disease modules or pathways. Here we develop an approach to systematically identify broad-spectrum drugs that target proteins exploited by multiple human disease pathways (Fig. 1a). Frequently, multiple infectious pathogens or toxins that negatively affect hosts by different mechanisms exploit the same host pathways^3^. This notion raises the prospect that multiplex approaches may lead to the discovery of broadly active and host-oriented infectious disease countermeasures that target host functions exploited by multiple pathogenic agents. We use genetics and a new drug screening methodology to identify and characterize the previously approved drug Bithionol as an inhibitor of host caspases, which reduces pathogenicity of a wide range of pathogenic agents, including ricin, anthrax lethal toxin, *Botulinum* neurotoxin A, diphtheria toxin, *Pseudomonas aeruginosa* exotoxin A, cholera toxin, and Zika virus.

## Results

### Identification of host hub proteins exploited by multiple pathogenic toxins

**Figure 1:**
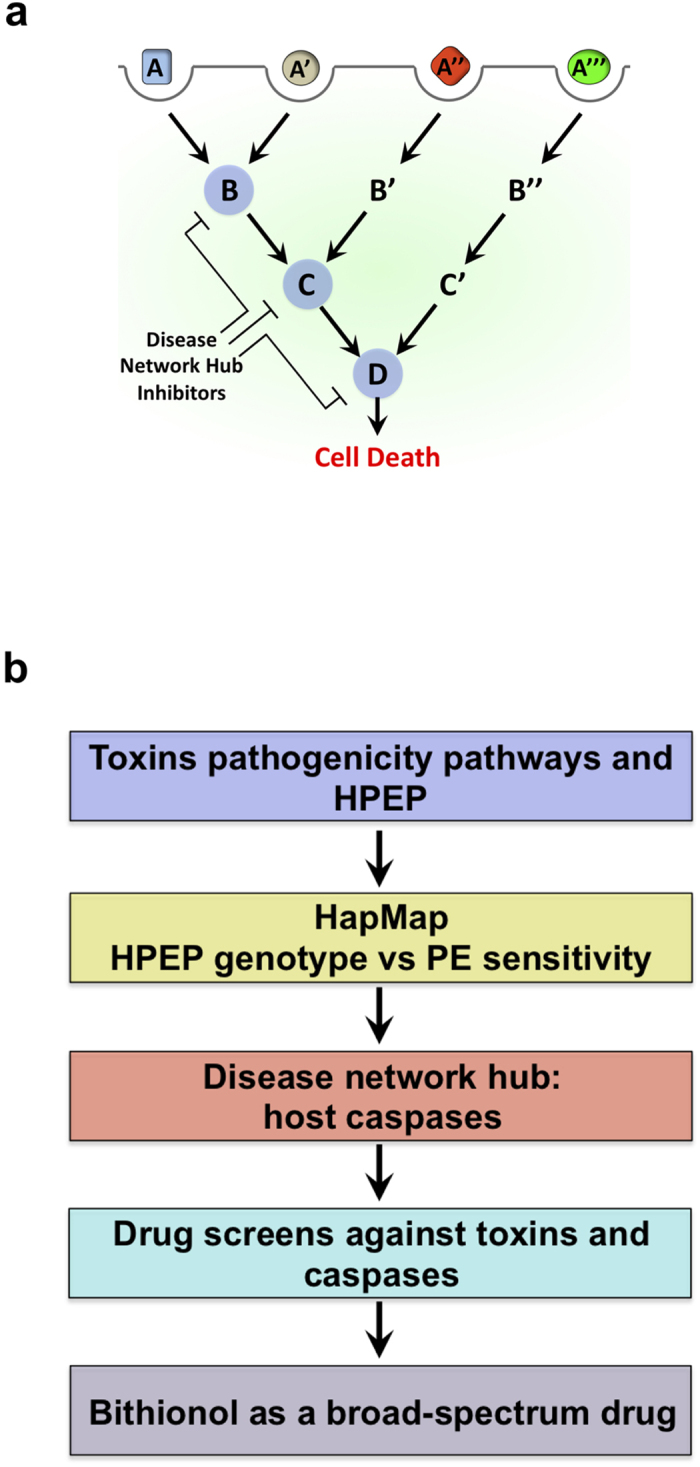
Inhibitors of hubs of human disease networks. (**a**) Depiction of the concept where multiple pathogenic pathways overlap, and hub proteins (blue circles) mediate multiple disease pathways. This model proposes a drug screen design to look for compounds that simultaneously inhibit the function of the hub proteins and reduce cellular lethality caused by multiple pathogenic pathways. (**b**) A depiction of the design of the current study. The known host pathways and host proteins exploited by pathogens (HPEP) are considered. HapMap cell lines are used to study the association between cellular sensitivity to *Pseudomonas aeruginosa* exotoxin A (PE) and genetic mutations in genes coding for proteins exploited by PE. Mutations in host caspases are associated with altered sensitivity to PE, and these proteins are defined as disease network hubs, which will be used as targets for the following drug screens. This approach yields the broad-spectrum, host-oriented drug, Bithionol.

Cytotoxic bacterial and plant toxins have evolved to exploit host proteins and cellular pathways that mediate the entry of those toxins into host cells and to induce cell-death. Although toxins exploit unique host pathways, these pathways are interconnected. While anthrax, diphtheria, and *Botulinum* toxins reach the cytoplasm from acidified endosomes, cholera, *Pseudomonas aeruginosa* and ricin toxins are transported into the cytoplasm through the host ER-associated degradation pathway^4^. These pathways interconnect at host “hub” proteins. Using one of those toxins, *Pseudomonas aeruginosa* exotoxin A (PE), we set out to identify such hub proteins by i) determining whether known genetic mutations in host proteins exploited by PE affect the sensitivity of host cells to this toxin, and ii) investigating whether these host proteins are also exploited by additional pathogenic agents. The protein hubs will be used as targets in drug screens in order to discover broad-spectrum, host-oriented, anti-pathogenic agent drugs (Fig. 1b).

### The effect of caspase mutations on the sensitivity of human B-cells to *P. aeruginosa* exotoxin A

**Figure 2:**
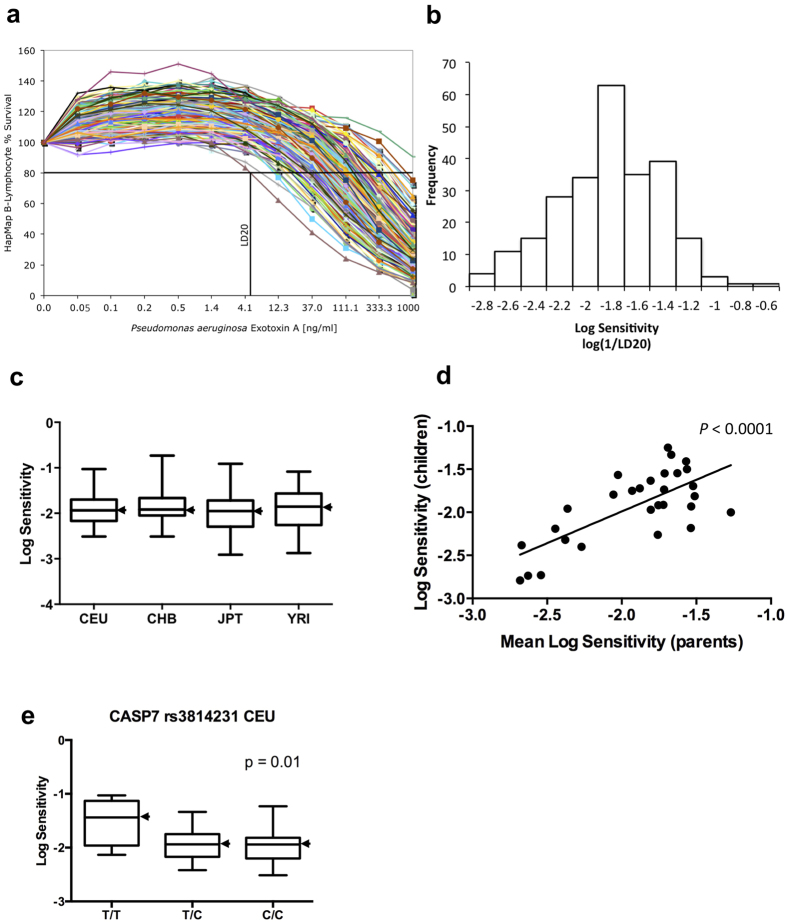
The effect of Bithionol on *P. aeruginosa* exotoxin A in human B-lymphoblastoid cells. (**a**,**b**) Human lymphoblastoid cells sensitivity to *P. aeruginosa* exotoxin A (PE)-mediated toxicity. (**a**) 234 B-lymphocytes were treated with PE at concentrations shown. Cell viability was determined by Alamar Blue assay (Materials and Methods) and is shown as the percentage of survivors relative to cells treated with PE alone. The LD20 calculation for the most sensitive cell line is shown as an example. (**b**) LD20 values (ng/ml of PE) were calculated and expressed on an inverse log10 scale. For calculations, PE sensitivity is defined numerically as 1/LD20. (**c**) Population-specific distribution of toxins sensitivities. CEU, YRI, JPT, and CHB denote European, Yoruba, Japanese, and Chinese Han, respectively. One CHB outlier is excluded. For each population, the black bar represents the median log sensitivity; the box extends from the lower to the upper quartile and the whiskers extend to the most extreme data point. (**d**) Heritability of log sensitivity in Yoruba trios. Plot of the log toxin sensitivity of the children against the mean log toxin sensitivity of the parents. The heritability is estimated as the slope (0.74) of the regression of the children phenotype on the midparent phenotype. (**e**) In CEU caspase-7 SNP rs3814231 associates with log PE sensitivity.

It has previously been shown that PE exploits several host proteins for its binding to and entry into host cells^5^ and initiates programmed cell death by inducing activities of host caspase-3, -6, and -7^6^. We investigated whether known mutations in host proteins exploited by PE associate with altered cytotoxicity of the toxin in cells from tissues that are naturally attacked by this toxin. The availability of human B-cells, which are physiological targets of PE^7^ through the HapMap Project^8^ has provided us with an opportunity to test whether mutations in host proteins that constitute the PE pathogenicity pathway affect the cellular sensitivity to this toxin. Our initial tests with cells from a few individuals revealed that their sensitivity to PE varies greatly. Remarkably, our further investigation of PE sensitivity of B-lymphoblastoid cells derived from 234 individuals in geographically and ethnically diverse human populations [87 Yoruba in Ibadan, Nigeria (YRI), 60 Utah residents with ancestry from northern and western Europe (CEU), 43 Japanese in Tokyo, Japan (JPT), and 44 Han Chinese in Beijing, China (CHB)] showed a prominent 200-fold difference in lethality to the toxin (Fig. 2a,b). The range in PE sensitivity, as measured by the dose required to kill 20% of the cells [log(1/LD20)], was similar in all four human cell populations (Fig. 2c). Analysis of toxin sensitivity in parent/children trios indicated that relative sensitivity to PE is a heritable trait (*P*-value <0.0001) (Fig. 2d).

**Figure 3:**
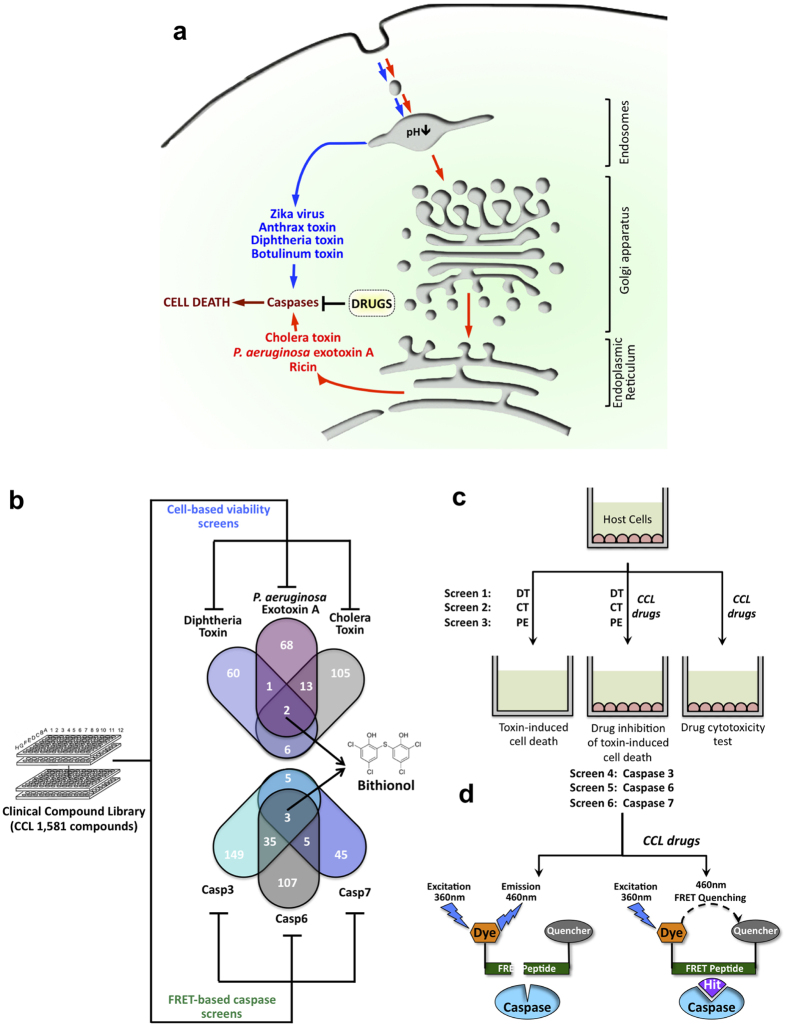
The use of the Clinical Compound Library (CCL) to screen for inhibitors of hubs of human disease networks. (**a**) Depiction of toxins as well as their pathways that induce caspase-mediated cell death. These toxins enter into host cytoplasm either from acidified endosomes or endoplasmic reticulum. Broad-spectrum anti-toxin drugs are screened to identify inhibitors of host caspases. (**b**) Overall approach scheme: CCL is screened by a multiplex approach that incorporates biochemical FRET and cell survival assays looking for drugs capable of simultaneously inhibiting host caspases-3/6/7 and reducing cytotoxicities of three bacterial toxins. The output of this approach is the discovery of broad-spectrum and host-oriented drug, Bithionol. (**c**) Schematic diagram of cellular screens to identify drugs that reduce cellular lethality induced by diphtheria toxin, *Pseudomonas aeruginosa* exotoxin A, and cholera toxin. Numbers are the distribution of inhibitors obtained in all screens. (**d**) Schematic diagram of parallel FRET screens to identify drugs that inhibit proteolytic reaction of caspases-3, -6, and -7.

The widespread and unimodal distribution observed for log(1/LD20) (Fig. 2b) is likely a result of a polygenic inheritance model, consistent with evidence that multiple host proteins mediate PE lethality^5^,^6^. To learn whether genetic variations in the genes encoding for these proteins account for any of the variation in sensitivity of B-cells to PE seen in Fig. 2a, we tested the association of numerous previously reported mutations with PE sensitivity. Caspase-7 and -3 mutations that were previously reported to associate with cancer and rheumatoid arthritis^9^,^10^,^11^,^12^,^13^ demonstrated a significant association with PE sensitivity in the individual HapMap populations (CASP7, rs3814231, CEU, *P* = 0.01; CASP7, rs2227309, CEU, *P* = 0.02; CASP3, rs4647601, rs4647693, and rs1049216, East Asian populations CHB and JPT combined, *P* = 0.04 for each SNP) (Figs 2e and S1). These results show that the activity of caspases affects host cell sensitivity to toxins, and that these proteins are potential therapeutic intervention points and targets for the following drug screens. In addition to PE, ricin and toxins of anthrax, diphtheria, *Botulinum*, and cholera induce programmed cellular death by activating host caspases^6^,^14^,^15^,^16^,^17^,^18^,^19^,^20^ (Fig. 3a). Therefore, a drug screen against hub caspases is of great interest, as these proteins are exploited by multiple pathogenic pathways, and caspase inhibitors can act as broad-spectrum drugs.

### A multiplexed cellular screen for CCL drugs that inhibit cytotoxic activities of bacterial toxins exploiting unique but interconnected host pathways

In an effort to identify existing drugs that might be repurposed as novel, host-oriented, broad-spectrum therapies, we screened a Clinical Compound Library (CCL)^21^ through multiplex-based drug screening (Fig. 3b). We searched for compounds capable of both (i) reducing cytotoxicities of diphtheria toxin, cholera toxin, and PE, and (ii) inhibiting proteolytic activities of host caspases-3, -6, and -7 exploited by these toxins in biochemical assays (Fig. 3b–d). In principle, a combination of biochemical and cellular drug screens could provide drug hits that reduce toxins’ cytotoxicities by inhibiting host caspases, and thus, this approach could simultaneously provide drug candidates and their protein targets.

We screened members of the CCL for the ability to reduce cell death of host RAW264.7 and C32 cells treated with PE, cholera toxin, or diphtheria toxin (Fig. 3c). At the indicated doses, between 30 and 50 percent of RAW264.7 cells undergo cell death within 12 hours for *Pseudomonas* and cholera toxins. For C32 cells, similar cell death was observed at 24 hours of exposure to diphtheria toxin under the experimental conditions employed. A “hit” in our screen was defined as an event where cells exposed to a drug increased cell survival by at least 20 standard deviations (~1% hit rate) above the survival of control cells treated with either toxin, and is not cytotoxic to cells in the absence of toxins. Events defined as “multiplex hits” interfered with cell killing by at least two toxins. The two multiplex hits that were identified as capable of reducing the cytotoxicities of all three toxins were Bithionol and Pyrogallol (Fig. 3b).

### A multiplex protein function-based screen for CCL drugs that inhibit proteolytic activities of host caspase-3, -6, and -7

**Figure 4:**
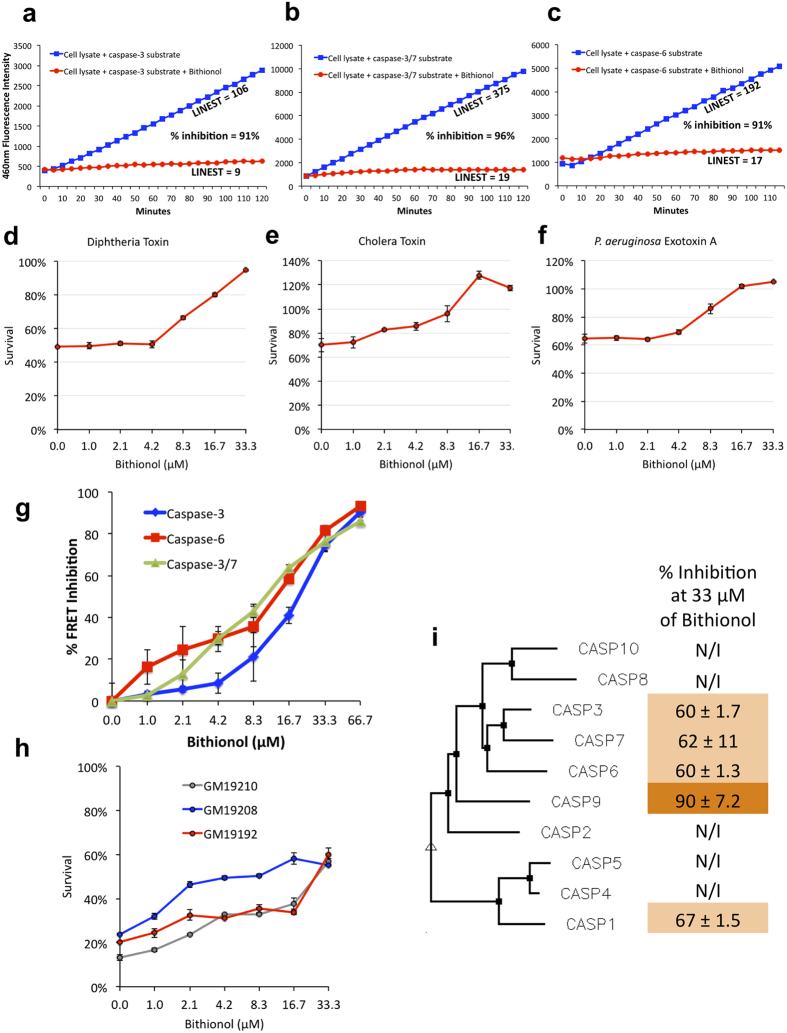
Bithionol reduces pathogenicity of toxins by inhibiting host caspases. (**a–c**) Bithionol inhibits caspases. FRET data showing fluorescence emission from two reactions, where caspase-containing cellular lysate cleaves fluorescently labeled substrate peptide without drugs, or in the presence of 33 μM Bithionol. FRET substrates were specific for cleavage by caspase-3 (**a**), caspase-3/7 (**b**), and caspase-6 (**c**). (**d–f**) Bithionol was tested for its ability to inhibit cytotoxicities mediated by toxins of cholera, diphtheria, and *Pseudomonas*. RAW264.7 cells were incubated with indicated doses of Bithionol for 1 hour, followed by 12 hours intoxication with *Pseudomonas* and cholera toxins. Diphtheria toxin was added to C32 cells for 24 hours. Cell viability was determined by MTT assay and is shown as the percentage of survivors relative to cells not treated with drugs. (**g**) Different concentrations of Bithionol are tested for their ability to inhibit caspase activity in cellular lysate of cells. Cells were pre-treated with *Pseudomonas aeruginosa* exotoxin A to induce caspases. FRET was done using substrates cleaved by caspases-3, -6, and -3/7. (i) Bithionol inhibits cytotoxicity mediated by *P. aeruginosa* exotoxin A in sensitive human B-lymphocytes. B-cells were seeded at 1 × 10^4^ cells/well on 96-well plates and were incubated with indicated doses of Bithionol for 1 hour, and then challenged with the toxin for 6 hours. Cell viability was determined by Alamar Blue assay and is shown as the percentage of survivors relative to cells not treated with drugs. (i) Bithionol inhibits caspases-1, -3, -6, -7, and -9. Bithionol was tested at 33 μM for its ability to inhibit FRET reactions of purified human caspases-1 through 10. Percent inhibition values are shown, and compared to activity of caspases untreated with Bithionol. Phenogram of ten human caspases, assembled by Multalin using Dayhoff alignment parameters, is used to demonstrate relative homology of caspases.

In parallel experiments, we screened the CCL for drugs that could inhibit the function of hub caspases-3, -6, and -7 (Fig. 3d) that mediate cytotoxicity caused by bacterial toxins used in our multiplex cellular drug screens (Fig. 3b). Caspase activities were induced in RAW264.7 cells by PE treatment. To screen and identify drugs that inhibit proteolytic activities of caspases we utilized a fluorescence-based FRET assay. Optimized substrate peptides for caspase-3, -6, and -3/7 proteolytic activities were used with a fluorogenic 7-amino-4-methylcoumarin group at the N-terminus and acetyl quenching group at the C-terminus. As a FRET substrate that is uniquely cleaved by caspase-7 hasn’t been identified, and since caspases-3 and -7 are close orthologues, we searched for caspase-7 inhibitors that could block proteolysis of a caspase-3/7 – specific substrate and not caspase-3 – specific substrate. After cleavage by caspase the fluorescence of AMC at 460 nm increases, while inhibitors of caspases prevent it. Compounds that showed greater than 80% (~1% hit rate) inhibition were defined as hits and selected for re-validation and further studies. Events defined as “multiplex hits” interfered with proteolytic activities of at least two caspases. Bithionol was one of the three multiplex hits identified as capable of prominently reducing the proteolysis of all three caspase substrates (Figs 3b and 4a–c). Since Bithionol was identified by both cellular and biochemical multiplex screens (Fig. 3b), we further investigated the efficacy of Bithionol and the breadth of its potential as a host-oriented, anti-pathogenic agent.

### Bithionol reduces the pathogenicity of a range of toxins by inhibiting host caspases

To investigate the potency of Bithionol, we first performed drug titration curves in host RAW264.7 and C32 cells. We demonstrated that Bithionol was able to reduce diphtheria, cholera, and *Pseudomonas* toxins-mediated cytotoxicities with an EC50 of 10 μM (Fig. 4d–f). We tested the effect of different concentrations of Bithionol for the ability to inhibit the proteolytic cleavage of substrates specific for cellular caspases-3, 6, and 3/7. We observed a linear dose-dependent caspase-inhibitory efficacy of Bithionol, with an IC50 of 21, 13, and 11 μM for caspases-3, -6, and -3/7, respectively (Fig. 4g). These results are consistent with anti-toxins EC50’s of Bithionol in cellular tests (Fig. 4d–f).

We also tested whether Bithionol reduces cellular sensitivity to PE in randomly selected PE-sensitive HapMap cells. We observed that the drug protected three cell lines treated with amounts of PE that are sufficient to kill 80% of cells (Fig. 4h). These results confirm the anti-toxin potential of Bithionol in host cells.

Humans have 10 well-characterized caspases that collectively form a pathway, often referred to as “the caspase cascade”, where caspases-3, -6, and -7 are the executioners of cell death and are activated by other caspases^22^. We tested the ability of Bithionol to inhibit activities of ten purified recombinant human caspase proteins, and we demonstrated that in addition to caspases-3, -6, and -7, Bithionol inhibited activities of caspases-1 and -9, while having no inhibitory effects on other caspases (Fig. 4i). Together, these results demonstrate that Bithionol is a direct inhibitor of a select subset of caspases, and that it reduces cellular sensitivity to toxins by targeting at least five host caspases.

### Bithionol inhibits cytotoxic activity of anthrax toxins

Anthrax toxins, the major virulence factors of the *Bacillus anthracis* bacterium, include an exotoxin protein complex consisting of a protective antigen (PA) and lethal factor (LF) that act collectively to damage host cells. PA binds to cellular receptors, while LF acts as a protease cleaving cytoplasmic MAPKKs^23^. Three additional host proteases mediate entry and lethality of anthrax toxin: furin, cathepsin-B, and caspase-1^14^,^23^,^24^.

**Figure 5:**
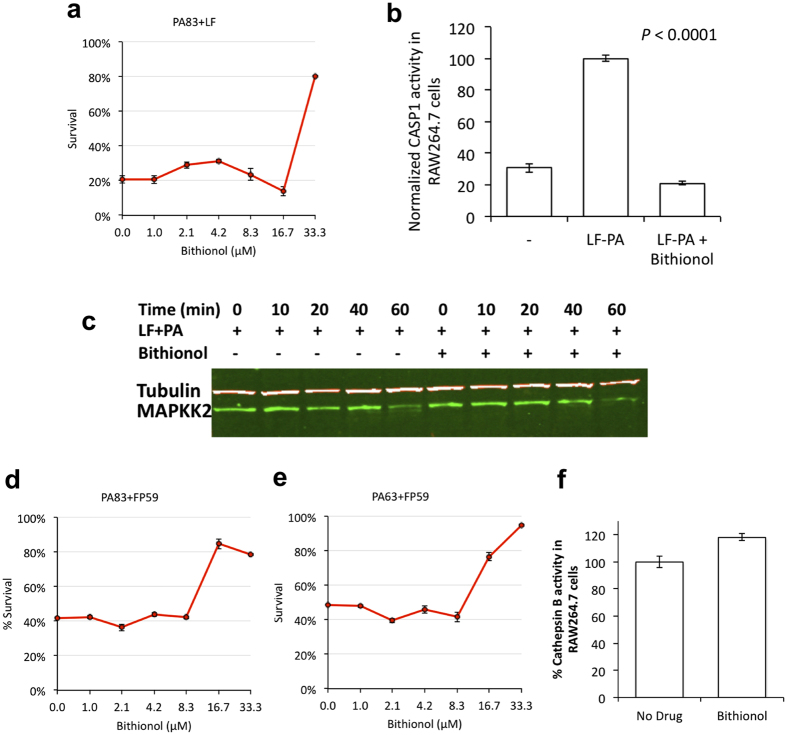
Bithionol inhibits anthrax toxin lethality. (**a**) Bithionol was tested for its ability to inhibit anthrax toxin-mediated cytotoxicity. RAW264.7 cells were incubated with the indicated doses of Bithionol for 1 hour, followed by 6 hours intoxication with anthrax toxin PA-LF. Cell viability was determined by MTT assay and is shown as the percentage of survivors relative to cells not treated with drugs. (**b**) Bithionol inhibits LF-PA–induced activity of cellular caspase-1. RAW264.7 cells were treated with LF-PA for 1 hour, and then treated either with 33 μM Bithionol or DMSO for 1 hour prior to lysis and determination of caspase-1 activity. The activity of caspase-1 was measured by FRET assay. (**c**) MAPKK2 immunoblotting showing that Bithionol does not block proteolysis of cellular MAPKKs by anthrax LF toxin. While MAPKK2 was cleaved in LF-PA treated RAW264.7 cells, treatment with Bithionol did not affect this process. RAW264.7 cells were incubated with Bithionol or DMSO for 1 hour before addition of vehicle control or 1 μg/ml PA + LF for up to 60 minutes. Cells were lysed and analyzed via immunoblotting with a MAPKK2–specific antibody. Tubulin was used as a loading control. (**d**,**e**) Bithionol reduces cell death induced by the hybrid toxin FP59, which has been widely used as an anthrax LF surrogate and contains the PA binding site of LF, as well as a toxin domain derived from PE. Bithionol-treated cells were found to be less sensitive to treatment with PA + FP59. PA was either in the native 83 kDa form (d), or used as 63 kDa–lacking 20 kDa Furin cleavage domain (**e**). RAW264.7 cells were preincubated with a titration of Bithionol for 1 hour, followed by a 6 hours intoxication with 0.5 μg/ml 83 kDa PA + FP59 or Furin processed 63 kDa PA + FP59. Cell viability was measured via MTT. (**f**) Bithionol doesn’t inhibit cathepsin B protease activity in RAW264.7 cells. RAW264.7 cells were treated with 33 μM Bithionol of DMSO for 1 hour prior to lysis, and determination of cathepsin B activity was assessed by FRET assay.

To test the ability of Bithionol to neutralize cytotoxic activity of anthrax toxin, we examined its effect on cell viability in LF-PA – treated RAW264.7 cells. While 80% of cells used for these assays normally undergo cell death within 6 hours of exposure to anthrax toxin, Bithionol provided substantial protection against LF-PA – mediated cell killing at 33 μM (Fig. 5a).

Caspase-1 activation, which occurs in LF-PA intoxication, was monitored using a FRET assay. While we observed an induction of caspase-1 activity upon LF-PA treatment in the absence of Bithionol, caspase-1 induction was not detected in Bithionol-treated cells challenged with anthrax toxin (Fig. 5b). This result confirms that Bithionol inhibits anthrax toxin cytotoxicity by at least inhibiting caspase-1 activity.

We investigated whether additional anthrax toxin pathway proteases are inhibited by Bithionol in live cells. By utilizing MAPKK immunobloting (Fig. 5c), a hybrid toxin FP59^25^ that enters host cells by utilizing PA, but kills cells by LF-independent mechanism (Fig. 5d,e), and cathepsin-B FRET assay (Fig. 5f), we demonstrated that Bithionol does not inhibit proteolytic activities of cellular LF, furin, and cathepsin-B.

### Bithionol inhibits ricin and Botulinum neurotoxin A - induced death *in vitro* and *in vivo*

**Figure 6:**
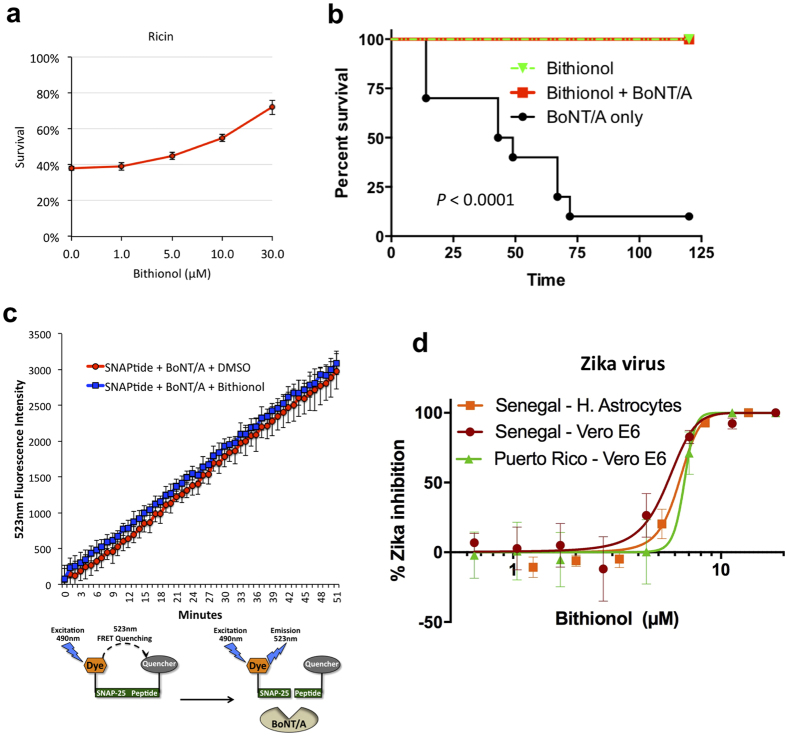
Bithionol acts as a broad-spectrum therapy. (**a**) Bithionol reduces ricin mediated cellular killing. Human K562 cells were incubated with indicated doses of Bithionol for 2 hours, and then challenged with ricin for 24 hours. Cell viability was determined by FSC/SSC flow cytometry. (**b**) Ten Swiss Webster CFW mice were treated with 6 mg/kg Bithionol in the presence or absence of botulinum neurotoxin serotype A complex (BoNT/A) by oral gavage. Animals were observed over 7 days. The Bithionol and BoNT/A survival curves are statistically different based on the Log-rank (Mantel-Cox) test, P < 0.0001. (**c**) Bithionol does not inhibit proteolytic activity of BoNT/A. FRET data showing fluorescence emission from two reactions, where 5 nM BoNT/A light chain cleaves fluorescently labeled SNAP-25 substrate peptide without drugs or in the presence of 33 μM Bithionol. (**d**) The ability of Bithionol to inhibit Zika virus (ZIKV) in host Vero E6 cells and astrocytes was measured by fluorescent microscopy. The virus-inhibitory EC50 concentrations were determined.

Ricin is another toxin known to induce host caspases-3, -6, and -7^15^,^20^. It reaches the mammalian cytoplasm through the retrograde transport route from the plasma membrane to ER via endosomes and the Golgi apparatus (Fig. 3a). Once in the cytoplasm, ricin inhibits cellular protein synthesis by cleaving a glycosidic bond within the large rRNA of the 60S subunit of eukaryotic ribosomes^4^. We tested the ability of Bithionol to reduce ricin - mediated cellular killing and observed that the drug was able to reduce toxin-mediated cytotoxicity with an EC50 of 10 μM (Fig. 6a).

*Botulinum* neurotoxin serotype A (BoNT/A) is a protease that translocates into the host cytoplasm from acidic endosomes, where it cleaves the synaptosome-associated protein, SNAP-25, and inhibits neurotransmitter release among neurons, leading to muscular paralysis^26^. BoNT/A has been reported to cause cellular caspases-3 and -7 - dependent apoptosis^16^.

After oral administration, Bithionol crosses the intestinal epithelium and is absorbed into the bloodstream in humans and many animals^27^. We evaluated the efficacy of Bithionol as a therapeutic agent during BoNT/A intoxication in Swiss Webster mice. Animals were given a lethal oral dose of BoNT/A complex in the presence and absence of Bithionol. Ninety percent of animals that received a lethal dose of BoNT/A without Bithionol died within 3 days of intoxication (Fig. 6b). All mice that were challenged with BoNT/A and treated with Bithionol at 6.0 mg/kg, survived without displaying toxin-associated symptoms, such as wasp waist and paralysis (Fig. 6b).

Since BoNT/A acts as a protease, we investigated whether Bithionol directly inhibits the proteolytic activity of BoNT/A by utilizing a FRET assay. An optimized SNAP-25 peptide with a fluorogenic FITC group at the N-terminus and DABCYL quenching group at the C-terminus was used as the substrate^28^. After cleavage by BoNT/A the fluorescence of FITC at 523 nm increases. We determined that Bithionol did not affect the proteolysis rate of the fluorescent substrate (Fig. 6c). This result shows that Bithionol protects mice by inhibiting host targets, rather than by inhibiting the toxin itself.

### Bithionol acts as a Zika virus inhibitor

In addition to pathogenic toxins, viruses are also known to propagate by activating host caspases and inducing programmed cell death^29^. Similarly to toxins, Zika virus (ZIKV) has been reported to lead to cell-death by inducing host caspase-3 and neuronal apoptosis during its propagation^30^,^31^. Moreover, caspases have previously been reported to cleave various viral proteins, affect viral protein localization, promote viral genome replication and viral assembly, and have been reported to be necessary for viral replication and propagation^32^,^33^.

Upon observing that Bithionol protects cells from caspase-inducing toxins, we hypothesized that Bithionol might also be able to inhibit the pathogenicity of the Zika virus. The strains utilized in this study were chosen to gauge the ability of Bithionol to inhibit Zika virus strains found within both ZIKV lineages. Both strains utilized in this study had low passage histories and had intact glycosylation sites. Furthermore, both strains were geographically and genetically divergent. Puerto Rico Zika strain, PRVABC59, is closely related to virus strains circulating in the New World including those strains isolated in Brazil and Guatemala. The African ZIKV lineage is ancestral to the Asian lineage; as such Senegal strain, DAK AR D 41525, was selected as it is a low passage strain that is mycoplasma free. We tested Bithionol’s ability to inhibit Senegal and Puerto Rico isolates of ZIKV in infected Vero E6 cells and human astrocytes. To detect infected cells, immuno-staining was performed using anti-Flavi-virus envelope protein antibodies. Bithionol inhibited the abundance of Puerto Rico ZIKV in Vero E6 cells with an EC50 of 6.7 μM as well as Senegal ZIKV in Vero E6 and human astrocytes with EC50’s of 5.5 and 6.3 μM respectively (Fig. 6d and Table S1). These data indicate that Bithionol is effective in inhibiting ZIKV in host cells.

## Discussion

In order to discover the inhibitors of pathway hubs that mediate the lethality of multiple bacterial toxins, we identified host caspases as hubs and focused the discovery process on therapies that are able to inhibit caspases and reduce toxins’ cytotoxicities. Collectively, utilization of both cellular- and biochemical-based multiplex drug screens provided us with i) a drug hit (Bithionol), ii) a drug-target (caspases), and iii) an assessment of breadth of spectra of the drug. This disease-network-based approach utilizing two diverse multiplex drug screenings, which we term “Disparate Dual Multiplexing”, could significantly shorten the time needed for drug-discovery followed by target-identification steps in a historically lengthy process. In the future, additional types of drug screens could be included in disparate multiplex-based drug screens, such as genetic-based, and immunology-based screens, thus expanding our approach to Disparate Triple, Disparate Quadruple (etc.) Multiplex drug screening.

Bithionol was previously approved to treat helminthes, and although the exact target within these species is not known, it is believed that the drug inhibits oxidative phosphorylation of parasites^34^. Our study has uncovered five additional Bithionol targets: caspases-1, -3, -6, -7, and -9, which by definition makes Bithionol a polypharmacological drug. Polypharmacological phenomena include a single drug acting on multiple targets of either a unique or multiple disease pathways. Just like in the current study, many of the polypharmacological approaches aim to discover the unknown off targets for existing drugs, and this approach is called “drug repurposing”^3^,^35^. Therefore, this raises the possibility that Bithionol targets additional non-caspase host proteins exploited by toxins. To our knowledge, no other drugs used in humans have been reported to inhibit caspases. In the future, Bithionol can be investigated for its efficacy to treat other non-infectious human diseases caused by detrimental activities of caspases, such as neurodegenerative diseases and inflammatory bowel disease.

Bithionol was replaced by the more effective anti-helminthic, Praziquantel^34^. Moreover, Bithionol was used as a bacteriostatic additive in cosmetic products, but was discontinued in 1967 due to photosensitizing effects^27^. Presently, Bithionol is still used to treat helminthic cases in some Asian and European countries and is included as an active ingredient in Fonergine^®^, used to treat mouth and throat disorders in Argentina^36^. The current drug discovery and development process can take many years for the successful introduction of a new drug into the market. Thus, the current *de novo* drug discovery and development paradigm is ineffective for dealing with rapidly emerging biological threat agents. Under such circumstances, drug repurposing may offer numerous advantages. Since Bithionol already has a well-established safety and pharmacokinetic profiles in patients and animals, as well as bulk manufacturing methods^27^, the drug could rapidly be made available for a new indication if a biological emergency was to occur. Most notably, it has been shown that after oral administration of 50 mg/kg of Bithionol in humans, the drug reaches serum concentrations that range from 225 to 480 μM^27^, which is on average 30 times higher than the EC50 achieved by our cellular tests (Figs 4, 5, and 6). This suggests that it is possible to achieve effective anti-toxin and anti-Zika Bithionol doses in blood by oral administration. In addition, Bithionol was i) used to treat cerebral paragonimiasis^37^ and is known to cross the brain-blood barrier^38^; ii) reported to cross the placental barrier^39^; and iii) was used to treat paragonimiasis in a population that included children and pregnant women^40^. All of these observations suggest that Bithionol is likely to achieve anti-Zika efficacy in humans.

## Experimental Procedures

### Chemicals and reagents

All bacterial toxins were purchased from List Biological Laboratories (Campbell, CA). FP59 was a gift from Stephen Leppla (NIAID). Ricin was purchased from Vector Laboratories. Clinical Compound Library (CCL) drug library was purchased from Johns Hopkins University Bloomberg School of Public Health. Bithionol was repurchased from Sigma-Aldrich.

### Cell culture and cell lines

RAW264.7 mouse macrophage and human C32 melanoma cells (ATCC CRL-1585) were maintained in DMEM (Sigma-Aldrich). Human B-lymphocytes were grown in IMDM (Invitrogen). Human K562 chronic myelogenous leukemia cells (ATCC CCL-243) were grown in RPMI 1640 Medium (Invitrogen). Vero E6 (ATCC CRL-1586) were maintained in MEM (Corning). Primary human Astrocytes (NHA-Astrocytes-AGM, Lonza, #CC-2565) were cultured in Astrocyte Basal Medium (Clonetics ABM, Lonza) supplemented with AGM SingleQuot Kit Supplement and Growth factors (CC-4123). All media were supplemented with FBS, penicillin, and streptomycin.

### Human B-lymphocytes sensitivity to Pseudomonas toxin-mediated lethality

Human B lymphocytes were treated with serial dilutions of *P. aeruginosa* exotoxin A for 48 hours. The viability of B cells was determined by Alamar Blue (AbD Serotec, BioRad) fluorescence, as described by the manufacturer. Each data point shown in Fig. 2a represents the average and SD of results from three wells. Cell viability is shown as the percentage of survivors obtained relative to cells in the absence of the toxin (100% survival). LD20 was calculated for each cell line. Statistical analysis and graphical presentation were performed using GraphPad Prism software. A *P* value <0.05 was considered statistically significant. Homozygous medians were compared by an unpaired t test. CHB and JPT, two East Asian populations, were pooled in order to obtain a more accurate estimate of effect size in the larger combined population. Because linear regression is sensitive to outliers, we removed one outlier in CHB, as the log sensitivity of this cell line couldn’t be determined within the toxin range tested.

### Cellular drug screens

RAW264.7 cells (10,000 per well) were seeded in 96-well plates 24 hours before the assay. Cells were treated with compounds for 1 hour, and then challenged with either 2 μg/ml PE or 4 μg/ml cholera toxin for 12 hours. As rodent cells are insensitive to diphtheria toxin, C32 cells were treated with 2 μg/ml of diphtheria toxins for 24 hours. Determination of RAW264.7 and C32 viability was performed by MTT assay. Cell viability is defined as the percentage of surviving cells obtained relative to cells treated with DMSO (100% survival).

### Caspases FRET drug tests

Caspases were induced in RAW264.7 by treating cells with 2 μg/ml of PE for 4 hours. Caspases were extracted and their activity was measured using Caspase Assay Kit (Sigma-Aldrich) with or without drugs.

The FRET reaction was performed in 96 well plates, and each reaction contained 16 μM substrate peptides (Peptides International) conjugated with a 7-amino-4-methylcoumarin group at the N-terminus and acetyl group at the C-terminus. The amino acid sequences of substrates were: DNLD for caspase-3, DQTD for caspases-3 and -7, and VEID for caspase-6. CCL compounds were tested at the final concentration of 33 μM, as pilot testing indicated that CCL screen at 16 μM would not yield a sufficient number of multiplex hits. Reactions were initiated by adding caspase – containing lysate to a final concentration of 6 μg/ml. Kinetic measurements were obtained at 37 °C every 5 minutes for 2 hours using a fluorescent plate reader. Excitation and emission wavelengths were 360 nm and 460 nm, respectively, with a cutoff wavelength of 365 nm. Rates of reactions were quantified by the Microsoft Excel LINEST function. A BioVision kit was used to test Bithionol’s ability to inhibit FRET reactions of purified recombinant human caspases-1 through 10. One unit of caspase was used in a single FRET reaction.

### Toxins treatments and cell viability assays

Cells (10,000 per well) were seeded in 96-well plates 24 hours before the assay. Cells were treated with Bithionol for 1 hour. RAW264.7 cells were challenged with anthrax toxins that include LF or FP59 and PA83 or PA63 (for 6 hours), PE (for 12 hours), or cholera toxin (for 12 hours) at 0.5, 2, and 4 μg/ml respectively. C32 cells were treated with 2 μg/ml of diphtheria toxin for 24 hours. Determination of cells viability was performed by MTT assay. B-lymphocytes cells were seeded in a 96-well plates at 10,000 cells/well 1 hour before toxin treatment, treated with Bithionol for 1 hour, and then challenged with 8 μg/ml PE for 6 hours. Determination of lymphocyte viability was determined by Alamar Blue (AbD Serotec, BioRad) fluorescence, as described by the manufacturer. Each data point shown for MTT and Alamar Blue assays indicates the mean ± SD value obtained in triplicate assays done in a representative experiment. At least three such experiments were carried out.

### MAPKK2 cleavage assay

N-terminal MAPKK2, anti-tubulin, and anti-PA antibodies were purchased from Santa Cruz Biotechnology. RAW264.7 cells were pre-treated with 33 μM of Bithionol for 1 hour. Following pre-treatment, the cells were exposed to 0.5 μg/ml of PA and LF at 37 °C for up to three hours in the presence of 33 μM of Bithionol. The cells were then washed with cold PBS five times and lysed with RIPA buffer containing a protease inhibitor mixture (Santa Cruz Biotechnology, Inc.). Cell lysates were quantified using the BCA protein quantification kit (Pierce) and loaded onto 4–12% denaturing gels (Criterion XT Precast Gel, Bio-Rad). After electrophoresis for several hours, the gel was transferred overnight to nitrocellulose membranes; membranes were probed with anti-MAPKK2 or anti tubulin antibodies. Quantitative Western blot analyses of the bands were accomplished using the Odyssey infrared imaging system (LI-COR Biosciences).

### Cellular cathepsin B and caspase-1 activity assays

Cathepsin B and caspase-1 activities in total cell lysates were determined using an InnoZyme cathepsin B activity assay kit (EMD Millipore) and caspase activity assay kit (BioVision), performed according to the manufacturers’ instructions. Cellular cathepsin B or caspase-1 activities with and without Bithionol were tested by pre-treating cells with 33 μM of Bithionol for 1 hour, followed by lysing cells and testing protease activities with fluorescently labeled specific substrates. Caspase-1 activities were induced by 1 hour pretreatment of cells with 0.5 μg/ml of LF + PA, and was compared to cells un-induced by the toxin. Fluorescence intensities indicating cathepsin B or caspase-1 activities were measured (Molecular Devices, Spectra Max 384 PLUS).

### Ricin treatment and cell viability assay

K562 cells were seeded at a density of 2 × 10^5^ cells/well in 24-well plates. Cells were pre-treated with Bithionol for 2 hours and 0.4 ng/ml ricin was added to the treated wells. Following the 24-hour ricin treatment, the percent of viable cells (within the live gate by FSC/SSC) was measured by flow cytometry using a BD Accuri C6 Flow Cytometer. Experiment was performed in duplicate for each condition.

### Mice intoxication studies

10 Swiss Webster (CFW) mice (6 weeks old) were treated with 0.125 mg/mouse Bithionol in the presence or absence of BoNT/A complex (3 μg/mouse, Metabiologics, Madison WI) in phosphate gelatin buffer (0.028 M sodium phosphate pH 7.0, 0.2% gelatin) by oral gavage. Animals were observed over a period of 7 days. Methods were carried out in accordance with approved guidelines. All experiments were performed in accordance with relevant guidelines and regulations. All animal experiments have been approved by the Western Regional Research Center IACUC. Euthanasia protocols follow recommendations established by the American Medical Veterinary Association Guideline for Euthanasia to minimize animal pain and suffering.

### BoNT/A light chain FRET assay

The reaction volume was 250 μl per well in 96 wells plate, containing 50 mM HEPES pH 7.4 containing 0.05% TWEEN 20, 5 μM SNAPtide (BoNT/A substrate peptide) conjugated with DABCYL and FITC (List Biological Laboratories, Inc), and 33 μM of Bithionol. Reactions were initiated by adding BoNT/A light chain to a final concentration of 5 nM. Kinetic measurements were obtained at 37 °C every 1 min for 50 min using a fluorescent plate reader. Excitation and emission wavelengths were 490 nm and 523 nm, respectively, with a cutoff wavelength of 495 nm. At least three such FRET experiments were carried out.

### Zika virus high content imaging infections assay

We utilized strains from both ZIKV lineages (African and Asian) to assess the potential for Bithionol to inhibit viral abundance. ZIKV strain DAK AR D 41525 was originally isolated from a pool of *Aedes africanus* mosquitoes in Senegal in 1984 (passage history: AP61#1, C6/36#1, Vero 3) and strain PRVABC59 was originally isolated from human sera in Puerto Rico in 2015 (passage history: Vero 6). Cells were pre-treated with Bithionol for 2 hours prior to infection. Vero E6 cells were exposed to ZIKV at an MOI of 0.5; astrocytes were infected with ZIKV at an MOI of 15. Infection was terminated after 48 hours by fixing samples in formalin solution. For visualization of Zika infection cells were treated for 1 hour with anti-Flavi-virus envelop protein monoclonal antibody 4G2, followed by anti-mouse IgG conjugated with Dylight488 (Thermo). Cells were stained with Hoechst (Life Technologies) for nuclei staining and with Cell Mask Red (Life technologies) for cytoplasm staining. Nuclei were stained with Draq5 (Biostatus) diluted in PBS buffer. Images were acquired on the Opera imaging plate reader (Perkin Elmer) and analyzed using Harmony and Acapella PE software. A detailed protocol is described in the supplemental file.

## Additional Information

**How to cite this article**: Leonardi, W. *et al*. Bithionol blocks pathogenicity of bacterial toxins, ricin, and Zika virus. *Sci. Rep*. **6**, 34475; doi: 10.1038/srep34475 (2016).

## Supplementary Material

Supplementary Information
