# Supplementary material for: Bithionol blocks pathogenicity of bacterial toxins, ricin, and Zika virus
Source: Sci Rep. 2016 Sep 30;6:34475. doi: 10.1038/srep34475 (PMC5043268; doi:10.1038/srep34475)
Supplement: Supplementary Information [file srep34475-s1.pdf]

Bithionol blocks pathogenicity of bacterial toxins, ricin, and Zika virus

William Leonardi,[1] Leeor Zilbermintz,[1] Luisa W. Cheng,[2] Josue Zozaya,[1] Sharon H. Tran,[1] Jeffrey H. Elliott,[1] Kseniya Polukhina,[1] Robert Manasherob,[3] Amy Li,[3] Xiaoli Chi,[4] Dima Gharaibeh,[4] Tara Kenny,[4] Rouzbeh Zamani,[4] Veronica Soloveva,[4,5,6] Andrew D. Haddow,[4] Farooq Nasar,[4] Sina Bavari,[4] Michael C. Bassik,[3] Stanley N. Cohen,[3] Anastasia Levitin,[1] and Mikhail Martchenko [1,*]

[1] Keck Graduate Institute, Claremont, CA 91711

[2] Foodborne Toxin Detection and Prevention Research Unit, Western Regional Research Center, United States Department of Agriculture (USDA), Albany, CA 94710

[3] Department of Genetics, Stanford University School of Medicine, Stanford, CA 94305

[4] US Army Medical Research Institute of Infectious Diseases (USAMRIID), Fort Detrick, MD, 21702

[5] Henry M. Jackson Foundation, Bethesda, MD, 20817

[6] Biotechnology High Performance Computing Software Applications Institute (BHSAI), Frederick, MD, 21702

*Contact: mikhail_martchenko@kgi.edu

SUPPLEMENTAL MATERIALS AND METHODS:

Zika virus High Content Imaging infections assay: Vero E6 cells were seeded in 384 plates (Aurora #1052-11130) at 2,000 cells/well in 40 µl of culture media for Zika virus infection assay. Astrocytes were lifted after culturing for 4 days using trypsin/EDTA (Lonza, #CC-5012) neutralized after 5 min by Trypsin neutralizing solution (Lonza, #CC-5002). Cells suspension was diluted with cultured media and plated at 20,000 cells/well in 96 well (Greiner, #655090). After 24 hours of incubation at 37°C with 5% CO_2 cells were treated with Bithionol to determine its antiviral activity. Ten serial dilutions of Bithionol in quadruplicate were added directly to the Vero E6 cell cultures using the HP D300 digital dispenser (Hewlett Packard, Palo Alto, CA) in 2-fold dilution increments starting at 100 µM at 2 hours prior to infection. Astrocytes were treated with 6 serial dilutions in 3-fold increment and starting at 50 µM. To generate Negative control column 3 in 384 well plate or row H in 96 well plates were treated with DMSO only (1%). The DMSO concentration in each well was normalized to 1% using an HP D-300 digital dispenser.

The ZIKV isolates used in this study were strain DAK AR D 41525 which was originally isolated from a pool of *Ae. africanus* mosquitoes in Senegal in 1984 (passage history: AP61#1, C6/36#1, Vero 3) and strain PRVABC59 which was originally isolated from human sera in Puerto Rico in 2015 (passage history: Vero 6). Two hours after treatment with compounds Vero E6 cells were infected with Zika virus at MOI = 0.5 (calculated for 4,000 cells/well, assuming one complete round of replication of Vero E6 cells at 15 ± 2 hrs after cell seeding). Human primary astrocytes were infected with Zika at MOI = 15 calculated for seeded density of astrocytes. Column 2 in 384 well plates

and row A in 96 well plates were left uninfected for positive control of inhibition. Infection was terminated after 48 hours by fixing samples in formalin solution.

Antiviral assays were done using a high content imaging technique for 384 or 96 well plates to quantify virus antigen production as a measure of virus infection. After pre-incubation of the cells for 30 min with blocking solution containing 1X PBS with 3% BSA (Sigma) supplements with 0.1% Triton X-100 (Sigma) the primary Anti-Flavi virus Group Antigen Antibody, clone D1-4G2 was diluted 1000-fold in blocking buffer (1X PBS with 3% BSA) and added to each well of the assay plate. The assay plates were incubated for 60 minutes at room temperature. The primary antibody was removed and the cells were washed 3 times with 1X PBS. The secondary detection antibody was an anti-mouse IgG conjugated with Dylight488 (#405310, Thermo Fisher Scientific, Waltham, MA) diluted 1,000-fold in blocking buffer and was added to each well in the assay plate. The assay plates were incubated for 60 minutes at room temperature. Nuclei were stained using Hoechst 33342 (H3570, Invitrogen), and the cytoplasm of cells was counter-stained with Cell Mask Deep Red (Cat #C10046, Thermo Fisher Scientific, Waltham, MA) diluted in 1X PBS. Cell images were acquired using Perkin Elmer Opera confocal plate reader (Perkin Elmer, Waltham, MA) using 10X Air objective to collect five images per each well. Virus-specific antigen was quantified by measuring fluorescence emission at a 488 nm wavelength, the nuclei staining was quantified by measuring fluorescence emission at a 400 nm and cytoplasm staining with Cell Mask at 640 nm. Analysis of acquired images was done using Acapella PE software. A “no virus” control (Column 2) and a “1% DMSO” control (Column 3) were included on each plate to determine the 0% and 100% virus infection, respectively. Hoechst 33342 signal

was used to generate nuclei mask and define each cell in the image. Cell Mask dye was used to better-defined area within the borders of the cells. The viral-antigen signal was compartmentalized within the cell mask. The intensity of signal in the 488nm channel collected from the control wells containing no virus infection was used to determine the threshold for low signal. Those cells that exhibited antigen signal higher than selected threshold were counted as positive for viral infection. Ratio of virus positive cells to total number of analyzed cells was used to determine percent of infection positive cells for each well on assay plate.

$$\%\,Infected\ cells\ \ = \frac{Positive\ Virus\ Number\ of\ Objects\ (s)}{Number\ of\ objects\ (s)} * 100$$

Two parameters, total the number of nuclei in each well and total percent of infected cells collected for each well on the plates were exported from PE Acapella into PE Columbus Image data storage system and GeneData Screener (Genedata Inc, Lexington, MA) software for further data management and analysis.

Data normalization was done on the plate level to allow comparison of the results from different experimental tests. The percent of inhibition of viral infection (% Activity or % Inhibition) was used to reflect decrease in percent of virus positive cells. The median of the values from the neutral control (NC) wells containing cells with viral infection and treated with DMSO were used as a minimal activity of inhibitor or 0% inhibition. The median of values from Blank control wells (BC) containing cells not infected with virus were used as a threshold for the low signal and values were converted into the maximum inhibition of viral infection or 100% inhibition. (S)- samples, values from the wells treated with compounds.

$$\% \, Inhibition = \frac{Median \, \%Virus \, positive \, (NC) - \, \%Virus \, positive(S)}{Median \, \%Virus \, positive(NC) - Median \, \%Virus \, positive(BC)} * 100\%$$

The values of % of infection and % of viability for each well was used in GraphPad Prism for curve fitting analysis using 2-4 parameter logistic (AC50, MAX, MIN, nHill) non-linear regression model to derive EC_{50} values corresponding to 50% of inhibition.

SUPPLEMENTAL FIGURE 1.

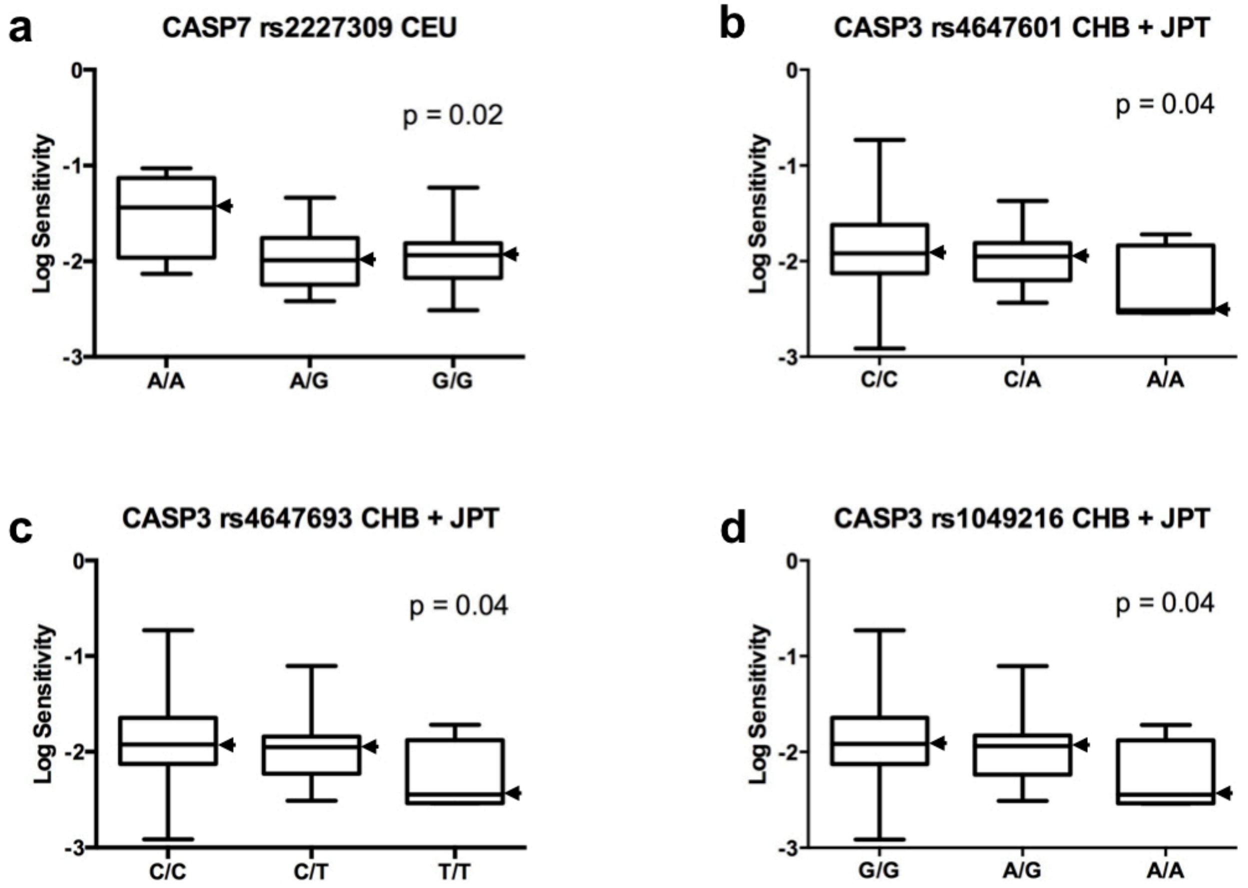

(A-D) Caspase-7 and Caspase-3 SNPs associate with log sensitivity of B-lymphoblastoid cells to *Pseudomonas aeruginosa* exotoxin A in indicated populations.

SUPPLEMENTAL TABLE:

Table S1: The effect of Bithionol on the pathogenicity of Zika Virus in cells. The ability of Bithionol to reduce the abundance of Zika virus in host cells (Vero E6 and human astrocytes) was measured by fluorescent microscopy. The 50% effective (EC50, virus-inhibitory) concentrations were determined.

Drug	Cells	Virus (strain)	EC50, μM	SD, μM
Bithionol	Vero E6	Zika (Puerto Rico)	6.66	1.15
Bithionol	Vero E6	Zika (Senegal)	5.52	0.29
Bithionol	Human Astrocytes	Zika (Senegal)	6.28	0.22
